# The Medical Education Partnership Initiative Effect on Increasing Health Professions Education and Research Capacity in Mozambique

**DOI:** 10.29024/aogh.14

**Published:** 2018-04-30

**Authors:** Emília Virgínia Noormahomed, Ana Olga Mocumbi, Mamudo Ismail, Carla Carrilho, Sam Patel, Alcido Nguenha, Roberto Badaro, Stephen Bickler, Constance A. Benson, Robert T. Schooley

**Affiliations:** 1 Department of Microbiology, Faculty of Medicine, Universidade Eduardo Mondlane (UEM), Maputo, MZ; 2 Department of Medicine, Division of Infectious Diseases, University of California, San Diego (UCSD), US; 3 Mozambique Institute for Health Education and Research (MIHER), Maputo, MZ; 4 National Institute of Health, Maputo, MZ; 5 Department of Pathology, UEM, Maputo, MZ; 6 Maputo Central Hospital (MCH), MZ; 7 Federal University of Bahia (UFBA), Salvador, BR; 8 Division of Pediatric Surgery, Rady Children’s Hospital, UCSD, US

## Abstract

**Background:**

Mozambique is an emerging lower income country (LIC) on the southeast coast of Africa. There are significant workforce shortages in medical and health professions in the country. Mozambique was one of 12 countries in Africa that was awarded a grant through the Medical Education Partnership Initiative (MEPI) in 2010. The overarching goal of MEPI Mozambique was to enhance the capacity of medical schools to train the medical and scientific leadership corps that the country required to facilitate the training of doctors and other health professionals, and thus to strengthen the national health system.

**Objective:**

The aim of this article is to provide an overview of MEPI Mozambique activities, its outcomes and successes, lessons learned, and how these have sustainably strengthened the health sector in the country.

**What Was Done:**

The Eduardo Mondlane University (UEM) formed a partnership with the University of California, San Diego (UCSD) to implement MEPI Mozambique. A range of activities in medical education, research capacity development, electronic connectivity and information technology, and developing relationships among medical education stakeholders, were performed.

**Outcomes and Effects:**

The activities and innovations introduced under MEPI became part of the daily routine in medical education in Mozambique, dramatically influencing attitudes and perceptions. Joint research with partners leveraged research capabilities. The creation of a research support center offered a mechanism to sustainably build on MEPI achievements. Scientific knowledge generated through research has been translated into practice and policy, and has improved the working environment for health professionals. The use of interactive communication technologies enabled the scaling up of training and research in sustainable ways, and created communities of practice.

**Conclusion:**

MEPI Mozambique developed transformational long-term partnerships between UEM, UCSD and other partners. These are changing the trajectory of medical and health professions education in Mozambique and creating sustainable capacity for research.

## Background

Mozambique is a country located on the southeast coast of Africa and is rich in natural resources. It spans 801,590 km^2^, has 2,750 km of coastline, and a population of approximately 28 million people, 69% of which live in rural areas [1]. The country is an emerging lower income country (LIC) that stands at 181 out of 185 countries in the United Nations Human Development Index, indicating a great need for human resource capacity development – especially in science, medicine and other health professions, as these are critically important for the sustainable development of the country [2].

When the country gained its independence from Portugal in 1975, there were 171 physicians and 1,960 nurses, a ratio of one physician per 63,000 people and 1 nurse per 579 people, respectively, in the healthcare system. The national health service coverage was less than 10% [3]. The health budget percentage rose from 3.3% in 1974 to 8.7% of the gross domestic product in 2015 [4,5]. Emphasis was placed on primary care training, and over a period of 30 years physicians were mainly deployed to primary care clinics with few receiving training beyond the basic medical degree [5,6].

To further exacerbate the scarcity of infrastructure and financial and human resources, the country went through 16 years of debilitating civil war from 1976 to 1992. This destroyed the country’s health services, leading to the closure of 1,113 clinics representing (48%) of the primary healthcare network between 1982 and 1990. Over 2,000,000 persons, including 880,000 children, were left without access to health care. The infant mortality rate rose from 200/1000 person-years in 1984 to 325–375/1000 persons-years, causing in excess of 494,000 childhood deaths between 1981 to 1988 due to the disruption in rural vaccination programs, risks of attack, fuel shortages for the mobile teams, etc [7]. The civil war also siphoned resources away from higher education, including the training of physicians and other health professionals.

The only existing medical school at the time, the UEM Faculty of Medicine (FoM), admitted native Mozambican students. However, resource constraints and faculty shortages limited physician graduates to fewer than 25 per year. Postgraduate medical education was virtually halted, except for a small residency program at UEM’s affiliated teaching hospital, Maputo Central Hospital (MCH), which increasingly compromised the medical leadership at the UEM FoM and at the Ministry of Health (MoH) [8]. Doctoral training occurred outside of the country through bilateral government cooperation but, on returning, these graduates encountered poor working conditions lacking infrastructure, equipment and finances to apply their expertise [9]. Furthermore, low salaries contributed to an internal brain drain from the public to the private sector, which pays three to four times more than public institutions. From 1980 to 2006, 25.5% (181) physicians in Mozambique left the public sector, of which 62.4% (113) continued working in-country and 37.6% (68) emigrated from Mozambique [10,11].

Since the 1992 peace accord, Mozambique has experienced stability and steady growth, and has one of the most rapidly growing gross domestic products in Africa with an average yearly growth rate of 6.6% [12]. GDP per capita increased from $133 per capita in 1983, to $161.31 in 1992, $210 in 1999 and $622.24 in 2014 [13].

By 2010 Mozambique was experiencing a steady improvement in living conditions. However, health care continued to be substantially under-resourced. Only 500 of the 800 existing physicians were actively practicing medicine, and many of them (30%) were working in the south, predominantly in the capital city, where they were attached to MCH and to the MoH headquarters.

Infectious diseases, including HIV, tuberculosis and malaria, continue to be the major causes of morbidity and mortality in Mozambique. In 2010 Mozambique’s adult HIV seroprevalence rate was approximately 12.5%, placing it among the worlds’ top ten. Other major medical problems include malaria and tuberculosis, accounting for 26% of all hospital admissions. HIV and/or tuberculosis account for approximately 60% of all admissions to MCH [14]. Schistosomiasis, waterborne bacterial diseases (*Salmonella, Shigella* and cholera), neurocysticercosis and leptospirosis also cause substantial morbidity. These contribute to Mozambique’s life expectancy at birth of less than 42 years (220th out of 223 in the world) [14,15].

Medical education, engineering and agricultural sciences have become a significant priority in view of the dire shortage of human resources in these fields. In 2007, the government established two new public universities outside of the capital city Maputo; these are supported by UEM in the training of human resources. Lúrio University (UniLúrio) was established in Nampula in 2006 and Zambeze University (UniZambeze) in Beira in 2009. Both locations are in underserved regions of the country. Currently, these new medical schools have few faculty members and much of the teaching is done by expatriate faculty (primarily from Cuba) or by visiting UEM and other partner university faculty, specifically for postgraduate training [9].

Mozambique was one of 12 countries in Africa that were awarded a grant through the Medical Education Partnership Initiative (MEPI) in 2010. MEPI aimed to develop transformative models in medical education and to build research and bioinformatics capacity to dramatically and sustainably increase the training and retention of physicians and scientists where they are most needed [9,11,16]. UEM formed a partnership with the University of California, San Diego (UCSD) to implement MEPI in Mozambique [9].

MEPI Mozambique had four primary objectives:

1. Enhance the capacity of undergraduate and postgraduate medical education in the Department of Medicine (DoM) at MCH;
2. Develop research capacity by providing training and mentorship to specialists and residents to conduct locally relevant clinical, epidemiological and translational research;
3. Develop electronic connectivity and information technology at MCH, FoM, UEM and the two recently created medical schools in the Nampula (UniLúrio) and Tete (UniZambeze) provinces; and
4. Improve relationships among stakeholders in medical education in Mozambique, namely the MoH and the Ministry of Education (MoE), the UEM FoM, UniLúrio and UniZambeze, the Mozambique Medical Council (MMC) and MCH.

In addition to the programmatic award, UEM was granted a linked award, which aimed to identify the best strategies for building emergency and essential surgical capacity in rural areas of Mozambique and to increase capacity for surgical research at UEM [17].

The aim of this article is to provide an overview of the MEPI activities in Mozambique, the outcomes and successes, lessons learned, and how this has built sustainability for the health sector in the country.

## What Was Done–MEPI Mozambique Activities

### Medical education

#### Postgraduate residency training

Medical specialist training at the MCH was revitalized and restructured. Formal training and curriculum innovation were introduced. Curriculum renewal in the residency training program in Mozambique included competency-based medical education, detailed descriptions of learning objectives, core educational activities, performance metrics and an evaluation system, graduation requirements, and timelines for achieving each [8].

A residency exchange program was launched between UCSD and UEM programs to facilitate bilateral sharing of expertise, educational mentoring, and development of English language skills for Mozambican trainees. Mozambican residents completed three-month clinical rotations at UCSD and UCSD residents completed one-month rotations at MCH. A total of 25 Mozambican residents and 50 UCSD residents participated [8].

A system of firm chiefs, called *monitores clínicos* and functioning similarly to “chief residents” in the U.S. system, was introduced in the DoM, and involved 12 young specialists and residents. Its objectives were to train in teaching and research methods, and facilitate better monitoring and evaluation of post-graduate teaching and healthcare delivery [8].

A structured didactic program for medical students and post-graduate trainees was introduced, consisting of teleconferences, afternoon lectures, case discussions, journal clubs and practical clinical sessions. Logbooks, designed to record performance and progress of individual residents, were introduced to document the clinical experiences of the internal medicine residents [8].

The postgraduate residency initiatives had the following outcomes:

- Eighteen residents completed internal medicine specialty training between 2010 and 2014. This represents a fourfold increase from the 12 graduates from 1991 to 2000 and 13 from 2001 to 2010. An additional 19 residents will qualify in the next four years [8].
- The *monitores clínicos* system has contributed to an increased number of ‘associate’ instructors for both undergraduate and postgraduate students and has substantially improved the mentoring process.
- The firm chief program was extended to the Department of Pediatrics and the Department of Surgery. To date, 12 firm chiefs have received additional training.
- The structured teaching programs introduced within the Department of Medicine at MCH, the main UEM teaching hospital, contributed to improved undergraduate and postgraduate training, peer mentoring skills in bedside and didactic teaching, as well as healthcare delivery, with patients benefiting from expanded physician contact.
- The logbooks have become a requirement for all residency training at MCH.
- The bidirectional residency exchange program between UCSD and UEM residents, allowing up to three months of training experience, permitted residents to focus on areas of expertise not available at one or the other institution, and facilitated improved English fluency for Mozambican residents [8].

#### New master’s programs

**Figure 1:**
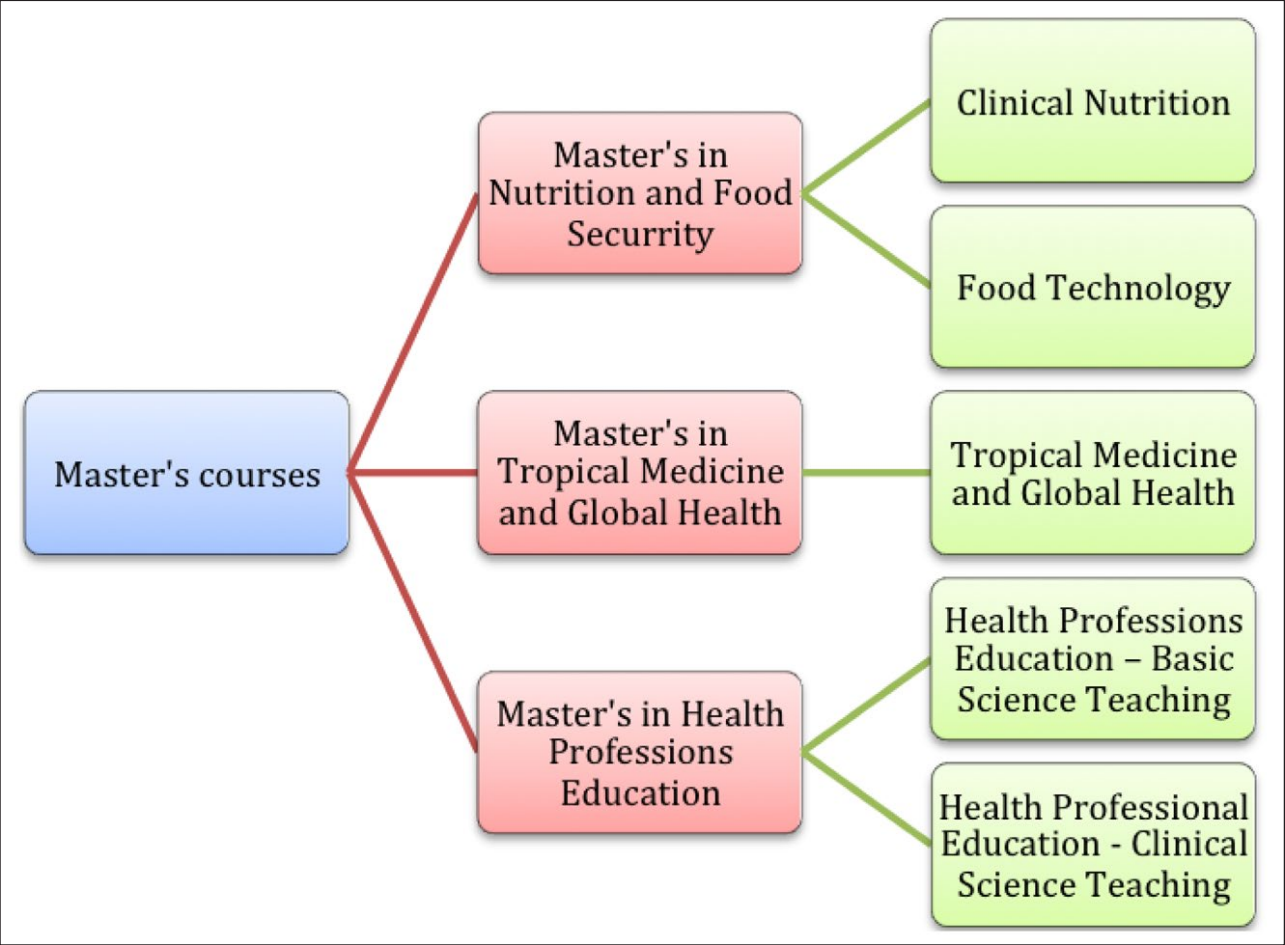
New Master’s Programs.

Three new master’s degree programs with five branches were established at UniLúrio (Figure 1). The aim of these programs was to increase the number of qualified medical educators at UEM, UniLúrio and UniZambeze; and to equip them with the improved teaching and assessment skills required to cope with the increased number of medical students being admitted into the schools. It is envisaged that these master’s programs will reduce the potential for brain drain from the country by providing opportunities for career development [9].

#### Continuing education

Continuing medical education and in-service clinical capacity building courses in the areas of HIV, nephrology, endocrinology, bedside teaching, ultrasonography and point-of-care technology were provided by UCSD specialists to the Mozambique residents. Twelve residents were trained in abbreviated point-of-care abdominal and thoracic ultrasound techniques. Clinical case discussions were held on a weekly basis through teleconferences between MCH and UCSD. Increasingly, presentations, learning materials and communication were in English with peers and mentors at USCD, positively fostering fluency in English [8].

Training to strengthen clinical skills related to pediatric trauma and burns was done through the MEPI surgery-linked award. A total of 61 health professionals attended a course on the management of pediatric burns. Residents from UCSD, Yale, Dartmouth and Harvard and surgeons from Vanderbilt visited MCH and participated in training courses at UEM that targeted pediatricians and surgical residents. These courses included didactic lectures, skills training, experience in clinical scenarios and a cadaver lab practical.

The linked award surgical training has re-invigorated the specialty of surgery in Mozambique, addressing the burden of disease both at the facility and community levels. Participation in the additional clinical training broadened the understanding and clinical skills of Mozambican surgical trainees and non-physician surgeons (surgery technicians) related to pediatric surgery, especially around care of burns and trauma. These trauma courses also included relevant information on HIV/AIDS. Diagnostic protocols and practical guidelines for pediatric surgical patients were developed and introduced to 103 health professionals.

#### Undergraduate education

Undergraduate health professions education benefited from the MEPI through the introduction of resources, including a “virtual” medical library, the laboratories for Infectious Diseases and Pathology, and the bioinformatics infrastructure that have been built with MEPI support as described below [11]. Clinical undergraduate teaching was strengthened by enhancing ward teaching sessions and training in the clinical applications of ultrasound, methods of clinical teaching, evidence-based healthcare practice, and research skills. In total 1,093 undergraduate students and 554 interns completed rotations on the four Internal Medicine wards participating in the firm chief system.

### Research capacity development

Training programs in research methods, grant proposal and manuscript writing, and human subject protection for research were organized by UEM for faculty, firm chiefs and postgraduate trainees at UEM and UniLúrio to address low local research productivity owing to lack of technical competencies (Table 1). These programs were also formally integrated into the postgraduate training program [8].

**Table 1:** Research Capacity Development Courses.

Courses	Number of Courses	Total Trained

Human Subject Protection for Research	5	98
Good Clinical Practice	2	58
Good Laboratory Practice	1	15
Manuscript Writing	4	81
CREST* – adapted from UCSD program training (4 modules)	4	51
CREST Seminar	1	52
Research Methodology	5	101
Project Design (for faculty/bioscience areas, UEM)	1	30
Grant Proposal Writing Workshop	2	74
How to Develop a Grant Budget Workshop	1	30
Research Methodology for Undergraduate Students	1	40

* Clinical Research Enhancement through Supplemental Training.

Mentors from several clinical departments at UEM and collaborating institutions were identified and paired with mentees to develop research topics jointly for grant proposal writing. Monthly sessions were established for postgraduate trainees, faculties and investigators to present, discuss and develop research proposals and protocols, and to enhance research proficiency. Each session was attended by an average of 30 persons [8].

Laboratory infrastructure and equipment upgrades were completed in the Department of Microbiology and the Department of Pathology. Renovation of laboratories and additional training of the technical staff enabled the conversion of these facilities into specialized Infectious Diseases and Pathology Laboratory Centers [11].

A joint Institutional Review Board (IRB) for the UEM and MCH was established to accelerate human subject research review and will progressively phase out the National Ethics Committee as the IRB for research conducted at UEM and MCH [11]. The IRB has reviewed 256 protocols from March 2013 to December 2015. An eplatform for submitting research protocols for review is under development.

A research support center, the Mozambique Institute for Health Education and Research (MIHER) (www.miher.org), was created to provide administrative and fiscal management support to the scientific community of public universities in Mozambique and to identify funding agencies that might be interested in supporting research activities [11]. MIHER has administered 22 research projects, coordinated 45 short training courses on research and has supported the design and implementation of the three master’s curricula at UniLúrio (Figure 1). MIHER has also provided support on project design, implementation and manuscript writing. In addition, MIHER partnered with CURE International, a non-profit international organization to support the Club Foot Program together with the national Department of Orthopedics at the MoH that aims to treat children by means of less invasive techniques. Thanks to partnerships with 10 clinics located in Maputo, Xai-Xai, Beira, Chimoio, Tete, Quelimane, Nampula, Lichinga and Pemba, 1,600 children have been treated or are under treatment.

A total of 63 new research projects (30 at UEM; 33 at UniLúrio) were developed in priority areas, involving mentors and collaborators from UEM, UCSD, the Federal University of Bahia in Salvador, Brazil (UFBA) and the Institute of Hygiene and Tropical Medicine at the Universidade Nova de Lisboa, Portugal (IHMT UNL) (Table 2). Among them, 19 have received external funding from multiple international institutions. In the MEPI surgery-linked award, several projects have been developed that focus on determining the burden of surgical conditions and define the unmet need for surgical care, such as types of surgical procedures performed by the non-physician surgeons, clinical epidemiology of pediatric trauma and risk-adjusted outcomes of surgical patients [18,19,20,21].

**Table 2:** Research Capacity Development Metrics.

Research Subject	Number

Research projects developed at UEM & MCH	30
Research projects developed at UniLúrio	33
Research skills courses provided to residents and specialists at MCH	22
Modules of the UCSD CREST* curriculum completed	4
Publications (37 published, 1 in press; 1 book chapter)	39
Local manuals, brochures and newsletters	4

* Clinical Research Enhancement through Supplemental Training.

Thus far, 39 peer-reviewed publications have been produced (37 manuscripts were published, one is in press; and one book chapter has been completed), while four local manuals and brochures (including one on Ebola) have been produced and eight additional papers are in the writing process (Table 2).

It has been possible to translate research into practice and policy. For example, the study on tuberculosis (TB) prevalence in MCH healthcare workers resulted in establishment and reinforcement of TB screening for MCH workers, continuing education programs and increased awareness of HIV/AIDS and TB in hospital, reinforcement of the distribution of personal protective equipment, and installation of ultraviolet lamps in sectors at risk for the spread of *M. tuberculosis*. Other examples include the two projects to develop point-of-care diagnostic devices, namely point-of-care ultrasonography for diagnostic HIV management and point-of-care CD4 cell counting for management of HIV coinfections [22]. Bedside diagnosis of HIV complications, including rapid CD4 cell counts and ultrasound evaluation, has been implemented as a result of these research initiatives.

### Electronic connectivity and information technology

Since 2011, e-learning has played a major role in building education, research capacity and communities of practice through MEPI. Examples of the MEPI elearning programs and projects that have been implemented include:

1. Facilitating an interactive live case conference connecting undergraduate and postgraduate trainees and faculty at UEM, MCH with their counterparts at UCSD using voice-over internet protocols (VOIPs) and telecommunications to present and discuss clinical cases, conduct lectures, and develop audience response-type questions to assess medical knowledge [8];
2. Using a similar VOIP format to allow UEM faculty to conduct lectures for medical students and faculties at UniLúrio as part of the undergraduate and master’s degree programs; and
3. Implementing the CREST course (Clinical Research Enhancement through Supplemental Training), a curriculum adapted from UCSD using internet resources to upload and conduct lectures and to facilitate interactions between students in Maputo and faculty in San Diego [8].

The main investment in e-learning tools and technology has been in the training of students in the use of e-learning tools, developing distance e-learning and telecommunications platforms, and acquiring educational resources and faculty to support master’s courses at UEM and UniLúrio. Equally important has been the establishment of infrastructure, wireless and bandwidth capacity to implement videoconferencing, and deploying and utilizing mobile tablets, iPads and other electronic devices for accessing e-learning technology at the UEM, UEM-FoM, MCH and UniLúrio, as well as in the anatomy and parasitology laboratories [8,11,23]. Virtual libraries have been established at the UEM FoM, the Internal Medicine Wards at MCH, and at UniLúrio. These were equipped with 100 computers, two servers, and other peripheral equipment, hardware and software to ensure adequate wireless connectivity and bandwidth. Google groups have been established for residency discussions. A central health registry has improved the patient record-keeping system. Through the MEPI surgery-linked award, an e-learning room, similar to the one developed for internal medicine residents and students, has been established for the surgical residents and students at MCH. See Table 3 for a listing of equipment and connectivity installed.

**Table 3:** Electronic Connectivity and Equipment.

Internet connectivity between the FoM, MCH and the Internet gateway at EMU’s main campus improved, bandwidth increased from 25 to 50mB.
Wi-Fi installed in the Internal Medicine, Surgery and Pediatric wards, the Emergency Room, teaching and conference rooms, the FoM Laboratory, the Clinical Laboratory and the Medical School.
Forty-eight iPads and iPods provided to residents for point-of-care access to medical literature and to increase IT skills.
Three virtual libraries with 75 computers, internet connectivity, printer, photocopy machine, access to e-books and hard copy books installed at the UEM-FoM, the MCH Department of Medicine and Department of Surgery, and 25 computers at UniLúrio.

### Relationships among Medical Education Stakeholders

Based on their best understanding of Mozambique’s needs, team members from both UEM and UCSD jointly designed the aims of the MEPI. These aims remained aligned with policies and priorities of the Mozambican government and key local institutions, including the MoH, the MoE, the Ministry of Science and Technology, the MMC, UEM, UniLúrio and UniZambeze. This facilitated broad ownership by local stakeholders and ensured future sustainability of the activities in Mozambique. The development of local expertise to sustain the infrastructure for research and training was emphasized. Faculty and laboratory technicians from the collaborating universities jointly initiated research projects, and designed training in research methods, grant proposal writing and scientific writing [11].

A multidisciplinary group of faculties from UEM, UCSD, UFBA and the IHMT UNL was created to provide ongoing mentoring and research capacity development within Mozambican partner institutions [11]. A motivated and dedicated group of mentees has emerged to carry the effort forward. Prior to the MEPI programs, there was no structured curriculum for postgraduate training of specialists in internal medicine, pediatrics or surgery. The training activities and the research training courses implemented through the MEPI have been integrated as recurring activities, now led by Mozambican faculty rather than being provided as external “enrichment” courses, and now obligatory components of postgraduate medical training by the MMC [8]. By aligning these activities within the university with recently re-energized national regulatory and governmental entities such as the MMC and the MoH, the programs have attracted local investment to ensure sustainability [8].

A MEPI team member was appointed as the National Surgery Program Chief at the MoH, a positive development that will ensure the continuity of training and research activities in collaboration with the MoH. Three faculty members have been selected as Fogarty Global Health Fellows under the UCSD Global Health Institute award.

Communities of practice have been successfully established with internal and external stakeholders. At the national level, the relationship between UEM and MCH, between the Regulatory Councils of Mozambique, and between the MoH, the MoE and the Ministry of Science and Technology, have all been strengthened. Multiple collaborations with universities from the United States (UCSD, University of Alabama at Birmingham, Vanderbilt University), the University of Granada and the University of Barcelona (Spain), the Universidade Nova de Lisboa and the University of Oporto (Portugal) and the Munich Technical University (Germany) have further strengthened the community of practice [9,11].

South-South collaborations with resident exchanges and collaborative research have been established with the University of Zimbabwe, the Kilimanjaro Christian Medical Center in Tanzania, Stellenbosch University, the University of KwaZulu-Natal and the University of Cape Town in South Africa, and the Federal University of Bahia and Fundação Osvaldo Cruz (Fiocruz) in Brazil [9].

### The Outcomes and Effects of MEPI Mozambique Activities

The MEPI Mozambique program has substantially transformed medical education in the country. With the development of faculty members, physicians, laboratory technicians and administrative staff, the attitudes and perceptions of those involved underwent dramatic change. The activities and innovations introduced under MEPI became part of everyone’s daily routine, and this has continued beyond MEPI funding.

Ownership of the program was paramount to achieving the results presented above. Faculty members were motivated and empowered, not only to proceed with their teaching routines, but also to seek continuing education and develop and strengthen their research skills, thus improving their knowledge and skills, as well as the quality of training.

**Figure 2:**
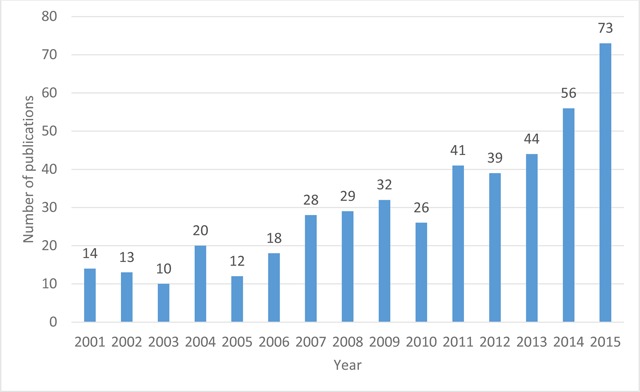
Number of Peer-Reviewed Publications at the FoM, EMU, and MCH, 2001–2015. Adapted from Noormahomed et al. (2013) [11].

Joint research by local lecturers and physicians, in collaboration with UCSD and other partners, has contributed to enhancing Mozambican research capacity. As Mozambique is a Lusophone country, linguistically linked to Portugal, the development of English language skills was encouraged and led to researchers applying for international research funding and producing publications in English peer-reviewed journals. This was important as African faculty that communicate in English publish more than those using other languages, and they and their universities are more frequently requested to partner with renowned academics and universities around the world. Research spending and English proficiency are strongly associated with publication output in the highest-ranked general medical journals [24]. The partnership not only increased the number of UEM FoM publications (Figure 2), but also the number of Mozambican first-authored publications from 29% in 2001–2010 to 38% from 2011–2013 [11]. The creation of the MIHER Research Support Center has offered a mechanism to continue to build on MEPI achievements, organizing short courses in didactic training, research and clinical skills courses, and joint research applications [11].

MEPI Mozambique has improved the working environment in the medical schools at UEM FoM, UniLúrio Faculty of Health Sciences, and MCH. The establishment of new master’s degree programs at UniLúrio allowed 45 new master’s degree students to complete their didactic training, finish dissertations and join the cadre of new teachers in the curriculum. The creation and refurbishment of libraries and informatics rooms at each institution provided students and residents with space to discuss clinical cases, and to access medical literature and scientific journals. Funds generated through research grants awarded to Mozambican faculty members provided incentives for them, residents, administrative staff and laboratory technicians. This stimulated the development of their careers, fostered professional satisfaction and motivation, and, in turn, resulted in retention of staff in the institutions [9]. Similar outcomes have been noted in other MEPI schools through improving the working environment and providing incentives influencing retention of health workers where they are most needed [25].

The MEPI has had a uniquely favorable impact on healthcare delivery within the Department of Internal Medicine at MCH. The post-graduate residency training activities, normally conducted exclusively in the mornings, were enhanced and extended to afternoon hours with increased teaching. Patients benefited from this additional physician interaction allowing admissions after 12 pm to be immediately assessed rather than waiting until the following morning [8]. Bidirectional teleconferences facilitating case discussions with residents from UCSD, teaching conferences delivered by UEM-FoM specialist faculty members, medical grand rounds, additional clinical seminars and a monthly clinical pathology session all contributed to improved patient care. This has allowed the Internal Medicine Department, previously considered to be in need of strengthening, to attract more residents, with enrollment increasing from 10 residents per year prior to the MEPI to 75 in 2012 [8]. The National Directorate of Health pronounced this as a model development, with some of these routines being replicated at the MCH Surgery Department. Surgical childcare was enhanced by partnering with programs such as the Club Foot Program, providing treatment for 1,600 children and facilitating the creation of club foot clinics in the country.

## Discussion

The long-term collaboration between Mozambican and US government institutions was the basis for a mutual interest in working together with the aim of addressing common issues in global health relevant to Mozambique. The economic and political stability of Mozambique offered an excellent environment for leveraging additional investments, as well as for engendering confidence in the country, further facilitating broader interactions and strengthening UCSD’s partnership with educational, healthcare, and government entities in Mozambique.

The strong leadership and ownership assumed by Mozambican researchers were very important for the success of this program as the components introduced into the program took into account the best knowledge and experience of local personnel, as well as the context in which the program was to be implemented. This was unlike some North-South collaborations where the objectives and implementation were driven by the northern partner [9,26]. This leadership facilitated the introduction of necessary change in local institutions, and was not seen as something imposed from the outside.

Another strength of this partnership was the donors’ flexibility, in this case PEPFAR and the NIH, which allowed the recipients to decide on the type of activities to be done, according to the terms of reference defined by the donors. Whenever necessary, the researchers proposed and made the changes necessary to the specific objectives in order to achieve the overall goals. The adaptation and simplification of financial and management systems according to the country’s conditions and needs included harmonizing fiscal and management systems with local policies and procedures, as well as continued investment in training of local administrators to ensure good accountability and management practices. For instance, MEPI funds could be used to seek out new partnerships in order to sustain and synergize activities, such as the Berlin Annual World Health Summit, the Consortia of Universities for Global Health, and the Prince Mahidol Award Conference. These principles contributed enormously to the success of the program [9].

The regular MEPI principal investigator council meetings that occurred every six months and the MEPI Annual Symposium allowed the sharing of experiences and expertise among different schools and countries. Communities of practice have been created and, whenever one of the schools involved was invited for other meetings it would take the opportunity to advertise MEPI activities on behalf of the other schools [9,27].

Through joint research partnerships, the use of distance learning for teaching and clinical care, and the establishment of master’s programs in an underserved region of the country, an environment has been established to retain faculty members and other health professionals where they are most needed. Funds generated through research grants and master’s student fees have provided support and incentives to faculties and administrative staff and improved their work environment. This in turn contributed to their career development and created professional satisfaction thus contributing to retention and further sustaining of MEPI goals [8,9,11].

Sustainability beyond MEPI funding has also been partially assured through continued joint research proposal development among MEPI and other key partners and continued activities of the MIHER Research Support Center to support fiscal management and seek additional funds. To date, MIHER has secured $5,716,175 for the next 5 years to be used for training, research and health care delivery activities to be carried out in the consortium universities and the Ministry of Health [9]. This program serves as a model for future projects designed to build health education and research capacity in a sustainable way.

A number of important lessons were learned through the MEPI program, for example:

- The implementation of e-learning at medical schools in Mozambique has been successful, showing that this is indeed possible within a resource-constrained environment.
- Master’s and doctoral programs can be developed with the support of bioinformatics tools, thus reducing inequities, transcending geographical barriers, and maximizing the few existing human resources.
- Low-cost informatics infrastructure and local knowledge capacity building can enhance both medical education and clinical care in resource-limited settings.

The program had its limitations and challenges. MEPI was a large and time-consuming program, which needed local and governmental champions, commitment, time and investment from many stakeholders. Limited resources and infrastructure curtailed capacity to replicate all the activities at both MCH and the participating consortium universities. The goals were ambitious and will have a bigger impact in the medium and long term. Mozambique and other LICs continue to be challenged by the poor global economic landscape and continue to rely mostly on external funding for research and some health programs.

To our knowledge, this program is unique in Mozambique as it emphasized local leadership and capacity building and empowered local experts to drive resource allocation decisions within UEM and the MoH for many years to come. Several sustainability strategies have been developed using the activities described in this article. The information technologies introduced and the training that has taken place to sustain these will help to reduce inequalities within Mozambique’s medical schools and allow open information exchange across universities and disciplines. MIHER will continue to provide research support to the academic communities in Mozambique, and its successes will attract additional grant funding and investment that will expand Mozambique’s footprint within the academic community of African medical schools [9,11]. The ultimate outcome will be to retain talented physicians and researchers within the Mozambican health care system.

## Conclusion

The overarching goal of MEPI Mozambique was to develop capacity for medical schools in Mozambique to produce the leadership required to advance the training of physicians and other health professionals, thus strengthening the national health system. Transformational long-term partnerships between UEM, UCSD and other partners have been developed, which are changing the trajectory of medical and health professions education and sustainable capacity for research in Mozambique. Some of the scientific knowledge generated through MEPI research has been translated into practice and policies. The program has leveraged the use of interactive communication technologies to scale up training and research in sustainable ways and to create communities of practice that will have a lasting impact.

## Figures and Tables

**Table:** 

UEM	Eduardo Mondlane University
FoM	Faculty of Medicine
UFBA	Federal University of Bahia, Brazil
HRSA	Health Resources and Services Administration
IHMT UNL	Institute of Hygiene and Tropical Medicine at the Universidade Nova de Lisboa, Portugal
IRB	Institutional Review Board
LIC	lower income country
MCH	Maputo Central Hospital
MEPI	Medical Education Partnership Initiative
MIHER	Mozambique Institute for Health Education and Research
MMC	Mozambican Medical Council
MoE	Ministry of Education
MoH	Ministry of Health
NIH	National Institutes of Health
PEPFAR	President’s Emergency Plan for Aids Relief
TB	tuberculosis
UCSD	University of California, San Diego
UniLúrio	Lúrio University
UniZambeze	Zambeze University
VOIPs	voice-over internet protocols
Fiocruz	Fundação Oswaldo Cruz
